# Editorial to the special issue *Neuronus*

**DOI:** 10.2478/v10053-008-0147-4

**Published:** 2013-12-31

**Authors:** Rob H. J. Van der Lubbe, Michał Kuniecki

**Affiliations:** 1 Cognitive Psychology and Ergonomics, University of Twente, The Netherlands; 2 Department of Cognitive Psychology, University of Finance and Management in Warsaw, Poland; 3 Psychophysiological Laboratory of the Jagiellonian University, Kraków, Poland

**Keywords:** right ear advantage, attention, EEG, beta band, visual word form area, disorders of consciousness, functional connectivity, default mode network, DTI, structural connectivity, MUC model, N400, neural oscillations, schizophrenia, insight, sleep, social cognition, lateralized power spectra, motion-based Simon effect, reaction time distribution, Adolf Beck

## Abstract

Did you visit the Neuronus conferences in the years 2012 and 2013 in Kraków? If not, then you certainly should have a close examination of this special issue including this introduction to at least have a glimpse of an idea of the highly interesting topics in the field of cognitive neuroscience that were presented at these conferences. If you were there, it is for sure a good choice to focus on this special issue as well, first to refresh your minds (we know our memories are far from perfect), but especially to see what happened with research of the presenters at these conferences.

## 

The *Neuronus* conference is traditionally situated in the beautiful town of Kraków in the south of Poland and is hosted at the Jagiellonian University. The organizers of this conference have a very good feeling for inviting interesting speakers, as you can see below. The conference is especially meant for students and young researchers in this highly interdisciplinary field but the conference is also well visited by older researchers. *Neuronus* has been supported by both the International Brain Research Organization (IBRO) and the International Research Universities Network (IRUN), a consortium of European universities founded by the Radboud University from Nijmegen in the Netherlands. In the last two years several hundreds of people participated in the conferences, and not only about 120 talks, but also approximately 135 posters were presented. As a consequence, the research reported in this special issue represents only a small part of the highly interesting materials presented at these meetings.

During the conference in 2012, it was announced that there would be a possibility of contributing to a special issue related to this meeting. It was indicated that this issue would be published in the journal *Advances in Cognitive Psychology* (another special issue devoted to a precursor of this conference appeared in the *International Journal of Psychophysiology*, Volume 85, Issue 1). After the conference, it appeared that 10 authors in total were able to submit their papers before the final deadline. After a rigorous reviewing procedure, four papers could finally be accepted for publication. Having attended to the memorial session devoted to Adolf Beck in 2013, we additionally decided to invite the contributors to submit a paper covering the major issues in this session, which after being submitted to a review procedure could be added as final paper of this special issue. Before shortly focusing on the contents of the papers in this special issue, we decided to inform you on some of the other very interesting contributions to these conferences, of which related materials were published in several journals.

In a plenary lecture on Friday the 20th of April 2012, Kenneth Hugdahl from the University of Bergen, Norway, informed us on a basic paradigm that he has been using for many years, which is based on an auditory laterality effect. Namely, when employing a dichotic listening task with different verbal stimuli simultaneously presented to both ears, the common observation is that the proportion of correct responses for stimuli presented to the right ear is higher as compared to the left. This effect is known as the *right ear advantage* and appears to be a robust indicator of a left hemispheric specialization for speech perception. In the same year, Hugdahl and colleagues (2012) published a paper in which the common right ear advantage was used in a more applied setting. Namely, they employed their paradigm to check the hypothesis that auditory verbal hallucinations can best be described as deviant speech perception. They argued that a consequence of these hallucinations would be that they also interfere with the processing of speech. This might show up in reduced sensitivity to external speech sounds when presented to the right ear, but not when presented to the left ear. Indeed, acquired hallucination scores correlated with correct responses with verbal stimuli presented to the right ear but not when presented to the left ear. Specifically, a higher hallucination score showed a reduction in correct responses for right ear stimuli. These findings support the idea that auditory verbal hallucinations can best be viewed as aberrant speech perception due to a malfunction of language areas in the left hemisphere.

In the cognitive session on Saturday the 21st of April, 2012, Mateusz Gola informed us on the observation that the EEG (electroencephalographic) *beta band* may be indicative of top-down attention. This issue was further detailed in a recent paper by Gola and colleagues (Gola, Magnuski, Szumska, & Wróbel, 2013). Although the EEG beta band is mostly related to motoric processes, as beta power decreases while preparing and executing voluntary movements, motor behavior is often accompanied with attentional processes. As a consequence, it may be the case that part of the observed activity is related to attentional rather than motor processes. As several studies reported that performance on attentional tasks is deteriorated in elderly, this might also show up in changes in posterior beta power. In an earlier study by Gola and colleagues (Gola, Kamiski, Brzezicka, & Wróbel, 2012), it was observed that lower beta band power over occipital sites in elderly was accompanied with decreased performance, but these results might possibly be due to the use of different strategies. In the recent 2013 paper, these issues were controlled for by using an adaptive procedure that enabled maintaining a similar level of performance for different age groups. The elderly group was further separated in a group with high and low performance. Interestingly, when comparing EEG activity between correct trials and trials without responses, it was observed that power in the beta band was higher for correct trials for the young and high performing elderly group, but not for the elderly group with low performance. These observations suggest that high beta power is indicative of good attentive performance, but also that elderly participants with low performance may have deficits in the underlying processes.

During the cognitive session on hemispheric asymmetry of language and vision on the 20th of April, 2012, Marcin Szwed presented evidence for cortical changes induced by the acquisition of reading skills (for a related publication, see Szwed, Ventura, Ouerido, Cohen, & Dehaene, 2012). He showed that observation of normal words compared to scrambled words was associated with higher activity in the *visual word form area *(VWFA), which is located in the left ventral occipito-temporal cortex. Interestingly, comparing intact letters with degraded letters also resulted in higher bilateral activity in early visual areas including V1, V2, V3, and V4. This pattern of activation seems specific for observing orthographic materials as it was not detected in control conditions in which objects consisting of line drawings were compared with scrambled objects. Szwed suggested that the letter-specific effect in early visual areas stems from perceptual learning of the specific shapes comprising the alphabet, as we all have to read fluently and fast in nowadays societies. During the next *Neuronus* conference in 2013, Szwed gave us an update on his previous talk, by describing the results obtained from two epileptic patients with electrodes implanted in the VWFA. The neurons within VWFA were found to respond 10 times stronger to letter stimuli than to other visual stimuli like landscapes, objects, or faces. These results further confirm the view that acquiring a highly specific skill like reading has a major impact on the development of specific brain areas like the VWFA.

In 2013, a plenary lecture on Friday the 10th of May, was given by Adrian Owen on disorders of consciousness. In this talk, he focused on circumstances in which neuroimaging data can be used to infer consciousness in the absence of behavioral responses. In a paper by Fernández-Espejo et al. (2012), coauthored by Owen, interest was focused on the possible relation between changes in *functional* and *structural connectivity* in patients with disorders of consciousness. Research focusing on functional connectivity within cortico-cortical and thalamo-cortical areas of the default mode network shows reduced connectivity for patients in a vegetative state as compared with patients in a minimally conscious state, which might be related to structural changes. The latter issue was explored by using diffusion tensor imaging (DTI) analysis to assess the structural integrity of the default mode network. A large group of patients from several hospitals was examined, including patients classified as being vegetative or being in a minimally conscious state. By employing DTI, it was possible to reconstruct the thalamo-cortical and cortico-cortical pathways connecting the major parts of the default mode network. It was observed that patients with consciousness disorders showed a clear impairment in these pathways relative to age-matched controls. Most interestingly, it was also observed that severity of the consciousness disorder correlated with the impairment of the structural connections. These findings additionally mark the crucial role of the default mode network for the emergence of awareness. Furthermore, these findings suggest that reduced structural connectivity is likely to be related to a loss in functional connectivity.

Two other highly interesting plenary lectures were presented on Saturday the 11th of May, 2013. Peter Hagoort focused on data supporting the Memory, Unification, and Control (MUC) model as a neurobiological model of language. In a recent paper, Hagoort (2013) argued that this model overcomes the limitations of the old Wernicke-Lichtheim-Geschwind model, which assumes a strict division between frontal and temporal regions in language production and language comprehension, respectively. For example, one major problem of this old model is that damage in frontal areas not only deteriorates language production but also impairs language comprehension. The MUC model distinguishes a *memory* component in temporal cortex, and the angular gyrus in parietal cortex that encompasses the know-ledge representations that store information including phonological word forms, morphological information, and syntactic templates associated with nouns, verbs, and adjectives, and also so-called *semantic convergence zone*s. Frontal areas are thought to be crucial for *unification* operations by generating larger structures from the parts that are retrieved from memory. This unification process concerns syntactic information, semantic information, and phonological aspects. Finally, *control* operations seem crucial in various respects, for example, in selection of the appropriate target language, in turn taking in conversation, and in paying attention to the most relevant input, which are thought to involve the dorsolateral prefrontal cortex. Interestingly, in his paper, Hagoort (2013) proposed a new account for the N400 event-related potentials (ERP) component, which can be derived from the EEG that he related with his MUC model. Hagoort proposed that incoming information of words in temporo-parietal cortex, the memory component, has two major effects. First, there will be a local spread of activity that may underlie effects like semantic priming. Secondly, there will be activation along a long-distance path to frontal areas that subsequently is thought to send back signals to the same areas in temporo-parietal cortex thereby inducing a kind of reverberation between temporo-parietal and frontal areas. This reentrant activity induces another spread of activation implying that connections representing a given local semantic context will be strengthened. If subsequently a word is presented that fits within the previously activated context, the neuronal response will be small; this effect seems comparable to effects of stimulus repetition. However, if the word does not fit within the pre-activated context, this leads to a large neuronal response, a clear N400 with a left temporo-parietal locus. Thus, according to the MUC model, the inverse relation between semantic relatedness and N400 amplitude is due to a reverberatory circuit linking temporo-parietal and frontal areas. An implication of this model that as far as we (the authors of this introductory paper) know has not been tested, seems to be that deactivating frontal areas by a method like transcranial magnetic stimulation (TMS) would reduce the spreading of activation as reverberation will be reduced. This might therefore result in a larger N400 component for words ending literal expressions like *animal* in the expression *my dog is an animal* as compared to a control condition in which frontal areas are not deactivated.

The other plenary lecture on Saturday the 11th of May, 2013, was held by Wolf Singer. He argued that higher cognitive functions require the coordination of large assemblies of spatially distributed neuronal networks by temporally structured activity, which may not only be related to a co-variation in neuronal oscillations but also to phase relationships of these oscillations. In a related paper by Uhlhaas and Singer (2012), they focused on the relevance of neural oscillations in disorders like schizophrenia and autism. They followed the idea that functional networks in various settings require dynamic rerouting and coordination, which can be achieved by modulating the coherence between distributed parts of the network. Furthermore, they argued that these dynamics are possibly disrupted in neuropsychiatric syndromes. A distinction was made between local oscillatory processes within the gamma band of the EEG that underlie the local encoding of information, while long-range synchronization couples more distinct brain areas, which might involve lower frequencies within the theta, alpha, and beta bands. With regard to schizophrenia, a reduction in gamma oscillations has been observed during the execution of cognitive tasks while other studies observed abnormalities in evoked activity in the theta and alpha band as well, which was interpreted as insufficient inhibition. During rest, however, schizophrenic patients showed increased activity in the gamma band. This might reflect increased autonomy of local parts of the networks and reduced global coordination. This increased local autonomy might, for example, explain why local beta- and gamma-band oscillations are increased when patients experience auditory hallucinations.

As indicated above, the current *Neuronus* special issue consists of five papers. The first paper, by Verleger, Rose, Wagner, Yordanova, and Kolev (2013), is a review paper that focuses on the role of sleep in gaining insight in a task that has an implicit regularity of stimuli. At a certain moment this regularity may become explicit (i.e., insight), leading to a major change in behavior that can easily be detected. It was shown that a night of sleep tripled insights relative to a baseline. Interestingly, this insight could be related to deep slow-wave sleep with a specific increase in the 10-12 Hz band of the EEG. In the second paper, by Niznikiewicz (2013), social communication is discussed in the context of social cognition, cold cognition (e.g., working memory, attention, etc.), and hot cognition (implying a role for emotions, intentions of involved people, etc.). On the basis of several ERP findings with emotion-related stimuli and varying effects of emotional state, Niznikiewicz argues that social cognition is accomplished by an interplay between fast sensory and later top-down processes. For example, N400 effects with emotional stimuli were shown to be modulated by mood. At the end of the paper, it was pointed out that several aspects of social communication are still far from understood, such as the function and underlying brain correlates of body language, leading to the conclusion that we are just beginning to unravel the crucially involved processes that shape social communication. The third paper, by Van der Lubbe and Utzerath (2013), presents a new method of analyzing lateralized EEG activity in an endogenous orienting paradigm with left or right attended locations by focusing on so-called *lateralized power spectra* (LPS), which can be computed by using wavelet analyses. It was revealed that commonly observed lateralized components in the cue-target interval were accompanied with lateralized changes in the theta, alpha, and beta bands. Furthermore, some effects were observed on the LPS that were not visible in the commonly employed analyses, which seemed related to either inter-individual differences or inter-trial fluctuations. In the fourth paper of this special issue, Strykowiec and Szczepanowski (2013) used reaction time distributions to examine whether the position-based stimulus response correspondence (SRC) effect, the classical Simon effect, and the motion-based SRC effect are likely to either or not have a common origin. On the basis of results of four experiments it was concluded that these phenomena are likely to be due to different underlying mechanisms as the position-based SRC effect showed a reduction for slower responses while the motion-based effect showed an increased effect for slower responses. In the fifth and final paper of this special issue, Coenen and Zayachkivska (2013) direct our attention back in the 19th century to Adolf Beck (1863-1942), who seemed forgotten for some time but actually can be considered as one of the founders of electroencephalography together with Richard Caton (1842-1926) and Hans Berger (1873-1941).

In conclusion, the recent *Neuronus* meetings in Kraków in 2012 and 2013 showed highly interesting talks on a variety of topics. With regard to the forthcoming issue in 2014, it can be assured that the organizers will again do their best to provide us with highly interesting plenary lectures, talks, and posters, and we would like to invite you to the next meeting in 2014 that will take place from 25th to 27th of April in Kraków. For more details and updates, see http://neuronusforum.pl
